# Genomic characteristics of a carbapenem-resistant NDM-1-producing *Aeromonas caviae* isolate from extraintestinal infection

**DOI:** 10.3389/fmicb.2026.1953006

**Published:** 2026-09-28

**Authors:** Zheng Bao, Zhenyu Mao, Yanye Tu

**Affiliations:** 1 Department of Clinical Laboratory, The Affiliated Li Huili Hospital of Ningbo University, Ningbo, Zhejiang, China; 2 Department of Clinical Laboratory, Xiangshan Red Cross Taiwan Compatriot Hospital Medical and Health Group, Xiangshan, Zhejiang, China; 3 Department of Clinical Laboratory, Ningbo Mingzhou Hospital, Zhejiang, Ningbo, China

**Keywords:** *Aeromonas caviae*, carbapenem resistance, extraintestinal infection, NDM-1 resistance gene, whole-genome sequencing

## Abstract

**Objective:**

This study aims to clarify the drug resistance characteristics and genomic background of bile-derived NDM-1-producing *Aeromonas caviae* (*A. caviae*) isolate from extraintestinal infection, providing a theoretical basis for the prevention and control of clinical infections.

**Methods:**

A bile-derived LHL25108073-*A. caviae* isolate (NCBI accession number: JBSUSV000000000) was chosen as the study focus. Through flight mass spectrometry, VITEK-2 drug susceptibility phenotype validation, and whole-genome sequencing, multi-point sequence typing (MLST), VFDB database, CARD database, PlasmidFinder 2.1 software, whole-genome alignment, plasmid synteny analysis, and plasmid conjugation assay were applied for an integrative analysis.

**Results:**

This isolate was identified to be ST2281, a sequence type rarely reported across clinical, environmental, and agricultural sources, with a genome size of 4,885,524 bp and a GC content of 61.27%, carrying one chromosome (4,687,937 bp) and one drug-resistant plasmid (197,587 bp). A total of 58 high-confidence virulence genes were detected, covering common flagella, polar flagella, lateral flagella, type IV pili, Msh pili, type VI/II secretion system (T6SS/T2SS), heme-uptake systems, Endotoxins, and hemolysin, forming multiple pathogenic mechanisms. Among 14 drug resistance genes, the carbapenem-resistant NDM-1 gene was highly consistent with the genotype, only sensitive to a few drugs such as amikacin and aztreonam, based on the results of drug susceptibility tests. The drug-resistant plasmid could not autonomously conjugate and transfer due to the loss of the oriT site and was untypable by conventional plasmid typing. This plasmid shared 99–100% homology in the resistance core regions with two NDM-1-carrying *A. caviae* plasmids, pK433-NDM (OK287926) from sputum in Taizhou, Zhejiang, and pFAHZZU2447_NDM (CP100393) from bile in Zhengzhou, with identical *bla*_NDM-1_ sequences. Genetic background analysis based on the genomes of 157 *A. caviae* isolates from China and neighboring regions identified that ST313 was the dominant sequence type (ST), which accounted for the largest proportion among in Chinese isolates.

**Conclusion:**

This study characterizes the molecular profiles of an extraintestinal, bile-derived NDM-1-producing *A. caviae* isolate belonging to ST2281, validates its abundant reservoir of virulence genes and comprehensive multi-drug resistance mechanisms, and further reveals the potential risk of NDM-1 dissemination via alternative horizontal gene transfer pathways, such as transformation and transduction. Genomic evolutionary analysis reveals that although the ST2281 isolate identified in this study shares certain phylogenetic relatedness with the prevalent ST313 lineage in China at the housekeeping gene level, whole-genome SNP-based phylogenetic analysis confirms a substantial genetic distance between them. ST2281 represents an independent and rare genetic lineage with limited global prevalence, which warrants heightened vigilance against its concealed transmission and potential clinical infection risks. Collectively, these results offer crucial genomic evidence for molecular tracing, targeted clinical antimicrobial therapy, and epidemiological containment of carbapenem resistant *A. caviae*.

## 1 Introduction

*Aeromonas caviae* (*A. caviae*), belonging to the genus *Aeromonas* of the family *Aeromonadaceae*, is a facultative anaerobic Gram-negative bacterium extensively in freshwater bodies, sediments, soils, and aquaculture water environments and colonizes the body surfaces and internal tissues of aquatic animals. It is a common opportunistic pathogen in aquaculture and clinical microbiological tests (Zhu et al., 2025). This bacterium has long been recognized as a low-virulence pathogen that predominantly infects immunocompromised individuals, the elderly, and patients with underlying chronic illnesses. Its hallmark clinical manifestations are present in the intestine, represented by acute gastroenteritis (Tang et al., 2023), accompanied by gastrointestinal symptoms such as abdominal pain and diarrhea, and the majority of mild infections achieve a favorable prognosis after symptomatic treatment. However, in recent years, with the extensive application of invasive diagnostic and therapeutic procedures, overuse of broad-spectrum antibiotics, and the growing number of elderly, critically ill, and immunosuppressed patients, the incidence of severe invasive infections caused by this bacterium is on the rise. Such severe infections encompass sepsis, urinary tract infections, suppurative infections of skin and soft tissue wounds, severe pneumonia, and abdominal bile infections (Jie and Shelat, 2021; Shin et al., 2022; Calvo Sánchez et al., 2025), and the infection risk is significantly increased, especially in special wards such as intensive care units (ICUs) and oncology department.

Bacterial virulence and drug resistance profiles are two core determinants of clinical infection prognosis. Existing molecular pathogenic studies have confirmed that the genome of *A. caviae* inherently encodes multiple functional virulence factors, such as aerolysin, thermolabile hemolysin, cytotoxic enterotoxins, adhesins, flagella, and outer membrane proteins (Sharma et al., 2022). Among these factors, aerolysin and hemolysin can directly lyse the host cell membrane (Zhu et al., 2025), disrupt the skin, intestinal, and vascular endothelial cell barriers of the body, and improve the invasion and immune escape ability of the strain *in vivo* (Jiang et al., 2025). In recent years, the most intractable clinical challenge lies in the fact that this opportunistic pathogen continuously acquires exogenous resistance genes under antimicrobial selective pressure (Zhou et al., 2024; Peng et al., 2025). Particularly, the emergence of carbapenem-resistant phenotypes (Luo et al., 2022), which further exacerbates its clinical threats (Hu et al., 2022; Gray et al., 2024).

Among diverse carbapenemases, New Delhi metallo-β-lactamase 1 (NDM-1) possesses broad hydrolytic activity and strong dissemination of antibiotic resistance (Ali et al., 2025), rendering it a priority carbapenemase subtype for clinical and public health surveillance. At present, epidemiological surveillance data at home and abroad have shown that clinically isolated NDM-1-producing strains primarily belong to *Enterobacteriaceae* bacteria (Huo et al., 2024), represented by *Klebsiella pneumoniae* and *Escherichia coli*. NDM-1-positive bacteria have also been detected in environmental water samples such as drinking water and sewage (Walsh et al., 2011). There is a well-established data system for studies on drug resistance transmission and molecular characterization of NDM-1-producing isolates from these two species. However, the genomic characteristics, resistance gene carriage patterns, and mobile genetic element structures of NDM-1-producing *A. caviae* remain poorly characterized, constituting a blind spot in the prevention and control of clinical nosocomial infections. Accumulating evidence indicates that NDM-1 producing strains from different bacterial genera and species exhibit significant species-specific differences in *bla*_NDM-1_ resistance gene vector types, upstream and downstream gene regulation pathways, and horizontal transmission mechanisms. Classic research findings indicate that *bla*_NDM-1_ resistance genes carried by *Enterobacteriaceae* bacteria are predominantly located on IncX3-type plasmids, which have high conjugation efficiency and can rapidly spread across different *Enterobacteriaceae* isolates (Guo et al., 2022). However, plasmid typing and genomic backbone structure can affect the transmissibility of mobile genetic elements. Therefore, the antimicrobial resistance dissemination profiles of *Enterobacteriaceae* bacteria observed cannot be directly extrapolated to guide the prevention, control, and treatment of nosocomial infections caused by NDM-1-producing *A. caviae*.

In addition, bacterial colonization and infection sites can also regulate the expression of bacterial virulence and resistance phenotypes. Previous studies on *A. caviae* have mainly focused on isolates of enteropathogenic bacteria from the digestive tract, which only need to adapt to the intestinal microenvironment to exert pathogenic effects. Different from the isolates of enteropathogenic bacteria, extraintestinal NDM-1-producing *A. caviae* must disrupt the intestinal mucosal barrier, invade the abdominal and biliary tracts, and adapt to a harsh *in vivo* microenvironment featured by high bile salt concentrations, hypoxia, and robust host immune surveillance (Hu et al., 2022). Genomic remodeling occurs in these isolates during the invasion and dissemination processes, which may endow them with unique virulence factors and molecular networks coordinating resistance and virulence co-expression. Whole-genome sequencing analysis can accurately identify the specific location of the *bla*_NDM-1_ resistance gene on the plasmid, analyze its surrounding genetic environment, and predict the risk of plasmid conjugational transmission; meanwhile, it can also explore virulence gene clusters related to extraintestinal invasion, providing data support for formulating precise infection control strategies.

Accordingly, we selected a carbapenem-resistant NDM-1-producing *A. caviae* isolate from clinical biliary bile samples as the research subject. High-throughput whole-genome sequencing and bioinformatic pipelines were utilized to analyze its full genomic backbone, annotate mobile genetic elements such as resistance/virulence genes and conjugative plasmids, and illustrate the genetic structure and transmissibility of the *bla*_NDM-1_-carrying plasmid. The findings will lay an important theoretical foundation for understanding the evolution and preventing the spread of NDM-1-producing *A. caviae*.

## 2 Materials and methods

### 2.1 Medical records

A carbapenem-resistant NDM-1-producing *A. caviae* isolate was found in the routine tests of clinical biliary bile samples in the microbiology laboratory of The Affiliated Li Huili Hospital of Ningbo University. A 73-year-old female patient was admitted to The Affiliated Li Huili Hospital of Ningbo University due to 1 month of chest tightness and abdominal pain, with a liver mass identified 2 days prior to admission. The auxiliary examination showed a significant elevation in the carbohydrate antigen 19–9 (CA19-9) level, suggesting a suspected liver cancer lesion. Pancreaticoduodenectomy was performed on October 6, 2025, and a carbapenem-resistant *A. caviae* isolate was acquired from biliary drainage fluid and cultured on October 13. This research was ratified by the Ethics Committee of The Affiliated Li Huili Hospital of Ningbo University (Ethics No.: Li Huili Hospital Ethics Review 2024-336).

### 2.2 Bacterial isolation, culture, and identification

Bacterial identification was achieved using the microbial mass spectrometry system EXS3600 (Zybio Inc., Chongqing, China). The *in vitro* drug sensitivity was examined using the VITEK-2 Compact system (bioMerieux, France). The Kirby-Bauer method was adopted for drug sensitivity verification, with the use of Imipenem-susceptibility disk (3768941; Oxoid, United Kingdom) and Gram-negative VITEK 2 Compact AST-GN335 (7753155503; bioMerieux). *Pseudomonas aeruginosa* ATCC27853 (GN335 quality control) for quality control was preserved in the biobank of The Affiliated Li Huili Hospital of Ningbo University. The susceptibility breakpoints were determined based on the US Clinical and Laboratory Standards Institute (CLSI-M45) standard.

### 2.3 Whole-genome sequencing

Genomic DNA was extracted through the sodium dodecyl sulfate (SDS) method, and DNA purity and integrity were detected by agarose gel electrophoresis, followed by a Qubit analysis for DNA quantitation. The qualified DNA samples following electrophoresis were randomly fragmented with a length of approximately 350 bp using a Covaris ultrasonicator. With the application of NEBNext^®^ Ultra™ DNA Library Prep Kit for Illumina (NEB, United States), the library was established through a series of steps, including end repair, A-tailing, sequencing adapter ligation, purification, and PCR amplification. After the library establishment, Qubit 2.0 was used for preliminary quantification, and the library was diluted to 2 ng/μL. Then, Agilent 2,100 was employed to detect the inserted fragments of the library. After the insert size was verified to meet the expected criteria, qualified libraries were sequenced on the PacBio RS II platform based on effective concentration and target data output. Whole-genome third-generation sequencing was completed by Sangon Biotech (Shanghai) Co., Ltd.

### 2.4 Bioinformatics analysis

#### 2.4.1 MLST homology analysis

The sequencing data were submitted to the PubMLST website^1^ to obtain the corresponding allelic numbers. The allelic numbers of six housekeeping genes (*gltA*, *groL*, *gyrB*, *metG*, *ppsA*, and *recA*) were queried against the database to determine the multilocus sequence typing (MLST) of the target strain.

#### 2.4.2 Virulence gene annotation

Virulence factors were identified by BLASTp alignment of the predicted protein sequences against the Virulence Factor Database (VFDB^2^). To ensure annotation reliability, only hits meeting the following criteria were retained: a percent identity ≥ 90% and a query coverage ≥ 95%. The retained genes were then integrated with their corresponding virulence factor functional annotation information to obtain the final annotation results.

#### 2.4.3 Antibiotic resistance gene annotation

Antimicrobial resistance genes were identified by BLASTp alignment of the predicted protein sequences against the Comprehensive Antibiotic Resistance Database (CARD^3^). The annotation was performed with an e-value cutoff of 1e-5, and only hits with ≥90% amino acid identity and ≥95% query coverage were retained. The retained genes were then integrated with their corresponding antibiotic resistance functional annotation information to obtain the final annotation results.

#### 2.4.4 Plasmid typing

The gene sequences were submitted to PlasmidFinder 2.1^4^ for plasmid type analysis.

#### 2.4.5 Whole-genome alignment

Nine isolates of *A. caviae* from different sources and isolation times, originating from mainland China and Taiwan, China, were downloaded from the NCBI database under accession numbers CP047981, CP071151, CP072326, CP084350, CP118442, CP119431, CP120942, CP121794, and CP177201. The chromosome sequence of LHL25108073 was used as the reference. Pairwise nucleotide comparisons were performed using BLASTn. High-scoring segment pairs of at least 100 bp were projected onto 5-kb reference windows; windows with at least 50% aligned coverage were classified according to mean nucleotide identity as ≥90%, 70–<90%, or 50–<70%. GC content, GC skew, comparative-genome similarity tracks, and selected high-confidence gene annotations were visualized using Circos.

#### 2.4.6 Plasmid synteny analysis

The data of two NDM-1-carrying plasmids from *A. caviae* were downloaded from the NCBI database for comparative genomic analysis: pK433-NDM (accession No. OK287926) from strain K433 isolated from sputum in Taizhou, Zhejiang, China, and pFAHZZU2447_NDM (accession No. CP100393) from strain FAHZZU2447 isolated from bile in Zhengzhou, China. A synteny map was generated using Easyfig software.

### 2.5 Plasmid conjugation assay

Rifampicin-resistant *Escherichia coli* EC600 was chosen as the recipient bacterium. Fresh *A. caviae* and recipient bacterium EC600 cultured overnight on Columbia blood agar plates were selected. The bacterial suspension (0.5 McFarland) was prepared using a turbidimeter. Next, 10 μL *A. caviae* and 20 μL EC600 were transferred to 10 mL Luria-Bertani broth medium and incubated overnight at 37 °C. After overnight cultivation, 1 mL of broth was diluted 10 times with normal saline. Then, 100 μL bacterial suspension was inoculated onto the screening plates containing 700 μg/mL rifampicin and 1 μg/mL imipenem. After overnight incubation at 37 °C, a single colony on the plate containing dual antibiotics was selected and identified using the fully automated microbial mass spectrometry system EXS3600, yielding *Escherichia coli*-positive results. Polymerase chain reaction (PCR), sequencing, and drug sensitivity tests were carried out to validate the results and determine the success of the conjugation assay, followed by plasmid conjugation prediction and identification using oriTfinder2. *Klebsiella pneumoniae* KP217360 (NCBI accession number: CP146099), carrying an NDM-conjugable plasmid, which was discovered by the research team in the preliminary study, was selected as the positive quality control bacterium for plasmid conjugation and is now preserved by our center (Tu et al., 2025).

### 2.6 Genetic background analysis

To analyze the genetic background of the isolate in this study, we downloaded the publicly available genome sequences of 157 *A. caviae* isolates from the NCBI Assembly database. Through the PubMLST database, we performed the MLST typing of all genomes, identified the sequence type (ST) of each isolate, and constructed a minimum spanning tree using GrapeTree tools based on the MLST allele profiles to analyze the regional ST distribution characteristics of the isolate in this study. To further improve the typing resolution, the third-generation sequencing genome assembly obtained in this study was chosen as a reference. Snippy was applied for genomic alignment and single nucleotide polymorphism (SNP) analysis on the screened regional isolates, and a core genome SNP matrix was subsequently constructed. Recombinant regions were then removed using Gubbins to exclude the confounding effects of homologous recombination on phylogenetic inference. A phylogenetic tree was established based on purified core SNP sites, and comprehensive annotation and genetic background analyses were completed by integrating STs, isolation time, and geographic origins.

## 3 Results

### 3.1 MLST types

In this study, the bile-derived NDM-1-producing *A. caviae* strain (designated LHL25108073-*A. caviae*, NCBI accession No. JBSUSV000000000) exhibited the following allelic profile: *gltA*_89, *groL*_751, *gyrB*_768, *metG*_595, *ppsA*_914, and *recA*_675, and was assigned to sequence type ST2281.

### 3.2 Virulence gene and drug resistance gene annotation

Third-generation sequencing of this isolate yielded a genome of 4,885,524 bp with a guanine-cytosine (GC) content of 61.27%. The genome consisted of a 4,687,937 bp chromosome and a 197,587 bp drug-resistant plasmid. By alignment against the VFDB database, 58 high-confidence virulence genes were identified in this isolate: common flagella (22 genes including *fliA*, *fliE*, *flgB*, and *cheV*); lateral flagella (15 genes including *lfiN*, *lfiQ*, *lfgG*, and *lafT*); polar flagella (2 genes: *motY*, *pomA2*); type IV pili (5 genes including *tapB*, *tapC*, and *tapT*); mannose-sensitive hemagglutinin (Msh) pili (1 gene: *mshE*); type VI secretion system (T6SS, 6 genes: *hcp*, *clpB*, *vasH*, *vasK*, *vgrG*, *vgrG1*); type II secretion system (T2SS, 4 genes: *exeE*, *exeF*, *exeG*, *exeI*); heme-uptake systems (1 gene: *amoA*); endotoxins (1 gene: *waaF*); and hemolysin (1 gene: *hlyA*) (Table 1).

**Table 1:** *Aeromonas caviae* VFDB database annotation results.

Virulence gene type	Virulence gene
Common flagella (*n* = 22)	*cheB-2*, *cheR-3*, *cheV*, *cheW-2*, *cheY*, *cheZ*, *fleN*, *fleR*, *fleS*, *flgB*, *flgF*, *flgG*, *flgI*, *flhA*, *fliA*, *fliE*, *fliF*, *fliG*, *fliI*, *fliM*, *fliN*, *fliQ*
Lateral flagella (*n* = 15)	*flgC*, *flgJ*, *fliP*, *lafC*, *lafT*, *lafU*, *lfgF*, *lfgG*, *lfgK*, *lfhA*, *lfhB*, *lfiE*, *lfiI*, *lfiN*, *lfiQ*
Polar flagella (*n* = 2)	*motY*, *pomA2*
Type IV pili (*n* = 5)	*tapB*, *tapC*, *tapF*, *tapT*, *tapW*
Msh pili (*n* = 1)	*mshE*
T6SS (*n* = 6)	*clpB*, *hcp*, *vasH*, *vasK*, *vgrG*, *vgrG1*
T2SS (*n* = 4)	*exeE*, *exeF*, *exeG*, *exeI*
Heme-uptake systems (*n* = 1)	*amoA*
Endotoxins (*n* = 1)	*waaF*
Hemolysin (*n* = 1)	*hlyA*

By alignment against the CARD database, 14 drug resistance genes were identified. On the chromosome, two β-lactamase genes were detected: MOX-3 and OXA-504. On the plasmid, 12 genes were identified, including the carbapenem resistance gene NDM-1; two β-lactamase genes (TEM-1, TEM-171); the sulfonamide resistance gene *sul1*; two macrolide resistance genes (*mphA* and *mrx*); the aminoglycoside resistance gene APH(6)-Id; the trimethoprim resistance gene *dfrA12*; the rifampicin resistance gene *arr-2*; the quinolone resistance gene QnrVC6; the bleomycin resistance gene BRP(MBL); and the disinfectant resistance gene *qacEΔ1*. The annotation results from the CARD database are depicted in Table 2. The results of the antimicrobial susceptibility test showed that the isolate was only sensitive to aminoglycoside amikacin, monocyclic β-lactam aztreonam, quinolone levofloxacin, and ciprofloxacin but resistant to all other tested agents, and the resistance phenotype and genotype profiles were basically consistent (Table 3). For both VFDB and CARD analyses, only annotations with ≥90% sequence identity and ≥95% query coverage were retained. Identity, query coverage, E-value, genomic location, database identifier, and annotation details for all 72 retained hits are provided in Supplementary Table S1.

**Table 2:** *Aeromonas caviae* CARD database annotation results.

Location	Drug resistance type	Drug resistance gene
Chromosome	β-lactamase (*n* = 2)	MOX-3, OXA-504
Plasmid	Carbapenem resistance (*n* = 1)	NDM-1
Plasmid	β-lactamase (*n* = 2)	TEM-1, TEM-171
Plasmid	Sulfonamide resistance (*n* = 1)	*sul1*
Plasmid	Macrolide resistance (*n* = 2)	*mphA*, *mrx*
Plasmid	Aminoglycoside resistance (*n* = 1)	APH(6)-Id
Plasmid	Trimethoprim resistance (*n* = 1)	*dfrA12*
Plasmid	Rifampicin resistance (*n* = 1)	*arr-2*
Plasmid	Quinolone resistance (*n* = 1)	QnrVC6
Plasmid	Bleomycin resistance (*n* = 1)	BRP(MBL)
Plasmid	Disinfectant resistance (*n* = 1)	*qacEΔ1*

**Table 3:** Drug sensitivity results of *Aeromonas caviae*.

Antimicrobial agent	MIC (μg/ml)	Susceptible (S) (μg/ml)	Intermediate (I) (μg/ml)	Resistant (R) (μg/ml)	Result
Amikacin^a^	4	≤16	32	≥64	S
Aztreonam^a^	2	≤4	8	≥16	S
Ceftazidime^a^	≥64	≤4	8	≥16	R
Cefepime^a^	16	≤2	4–8	≥16	R
Imipenem^a^	4	≤1	2	≥4	R
Meropenem^a^	8	≤1	2	≥4	R
Levofloxacin^a^	1	≤2	4	≥8	S
Ciprofloxacin^a^	0.5	≤1	2	≥4	S
TMP/SMZ^a^	≥320	≤2/38	-	≥4/76	R
Piperacillin/Tazobactam^a^	≥128/4	≤16/4	32/4–62/4	≥128/4	R

^a^Susceptibility testing reference guidelines: CLSI M45, 3rd ed., 2015, 16–18.

### 3.3 Whole-genome alignment

We generated a comparative circular chromosome map of LHL25108073 (4,687,937 bp) with selected high-confidence chromosomal resistance genes and virulence factors. The chromosome harbored the β-lactamase genes MOX-3 and OXA-504, together with selected virulence genes representing polar flagella, type IV and Msh pili, T6SS, T2SS, heme uptake, and hemolysin-associated functions. Compared with nine *A. caviae* isolates registered as CP047981 (Beijing, drainage liquid, isolation time: 2020-07-23), CP071151 (Wenzhou, unknown, isolation time: 2021-03-07), CP072326 (Henan, sputum, isolation time: 2022-06-27), CP084350 (Ningbo, bile, isolation time: 2021-10-10), CP118442 (Chongqing, unknown, isolation time: 2023-02-28), CP119431 (Taizhou, feces, isolation time: 2024-12-03), CP120942 (Zhuhai, urine, isolation time: 2023-04-05), CP121794 (Taiwan, China, unknown, isolation time: 2023-04-17), and CP177201 (Quzhou, hospital sewage, isolation time: 2024-12-03), whole-genome comparison revealed regions spanning ≥90%, 70–<90%, and 50–<70% nucleotide identity (Figure 1). Additional nucleotide-level validation identified full-length waaF homologs in all nine comparison genomes, each with 100% query coverage and 98.179–99.180% sequence identity. The three nearest transcriptional upstream ORFs were also present and retained conserved local synteny in all nine genomes (Supplementary Table S2).

**Figure 1:**
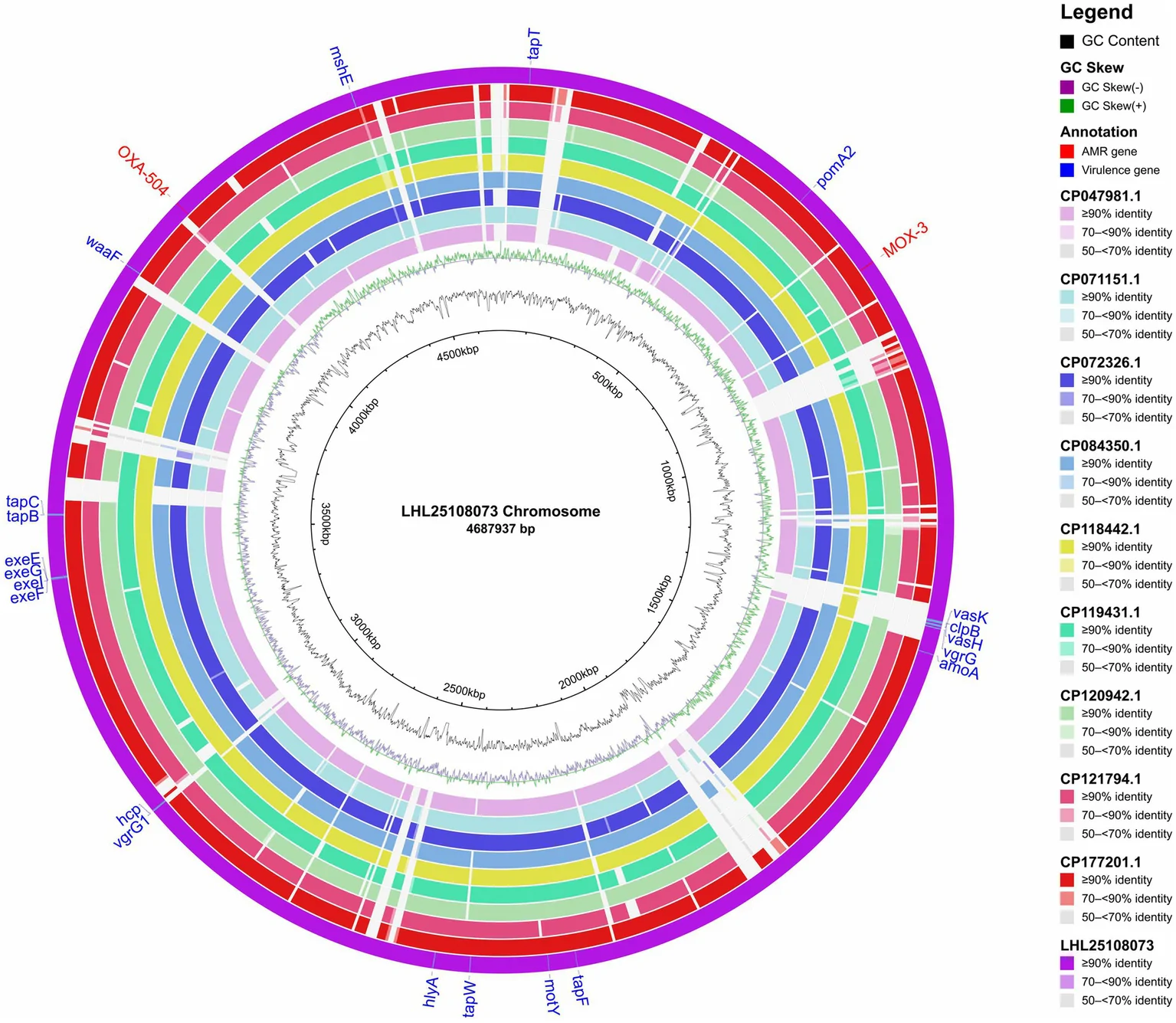
Comparative genomic Circos plot of *Aeromonas caviae* LHL25108073 and nine reference genomes. The center indicates the chromosome name and size (4,687,937 bp). Inner tracks show genomic coordinates, GC content, and GC skew. The outer colored tracks represent nucleotide similarity between LHL25108073 and nine *A. caviae* reference genomes (CP047981.1, CP071151.1, CP072326.1, CP084350.1, CP118442.1, CP119431.1, CP120942.1, NZ_CP121794.1, and CP177201.1), classified as ≥90%, 70–<90%, and 50–<70% identity. Selected high-confidence chromosomal antimicrobial resistance genes are shown in red, whereas selected high-confidence virulence genes are shown in blue. Only annotations meeting ≥90% sequence identity and ≥95% query coverage are displayed.

### 3.4 Plasmid conjugation assay and plasmid synteny analysis

The oriTfinder2 results showed that the drug-resistant plasmid, with a full length of 197,587 bp and a GC content of 61.27%, carried genes encoding Relaxase (*orf80*), type IV chaperone protein (*orf43* and *orf92*), as well as the T4SS system (*orf90*, belonging to the *virB1* family). However, this plasmid lacked the essential origin of transfer (*oriT*), rendering it theoretically incapable of achieving autonomous horizontal plasmid transfer between bacteria. This plasmid was inferred to be a non-conjugative plasmid, a conclusion concordant with repeated failure of plasmid conjugation assays (Table 4 and Figure 2). Moreover, the PlasmidFinder 2.1 software was unable to assign a replicon type to this plasmid.

**Table 4:** Annotation results of oriTfinder2 for NDM-1 resistant plasmids.

Type	Gene name
*oriT* region	–
Relaxase	*orf80*
Type IV chaperone Protein	*orf43*, *orf92*
Auxiliary protein	–
T4SS	*orf86*, *orf89*, *orf90*, *orf91*, *orf93*
ARG	*aph(6)-Id*, *bla*_TEM-1B_, *mph(A)*, *sul1*, *bla*_OXA-427_, *bla*_NDM-1_, *sul1*, *qacE*, *dfrA12*, *aac(3)-IId*, *bla*_TEM-1B_, *qnrVC1*, *ARR-3*, *qacE*, *sul1*
Metal resistance	*golS*, *golT*, *ncrA*, *merR*, *merT*, *merP*, *merA*, *merD*, *merE*, *merD*, *merA*, *merP*, *merT*, *merR*

**Figure 2:**
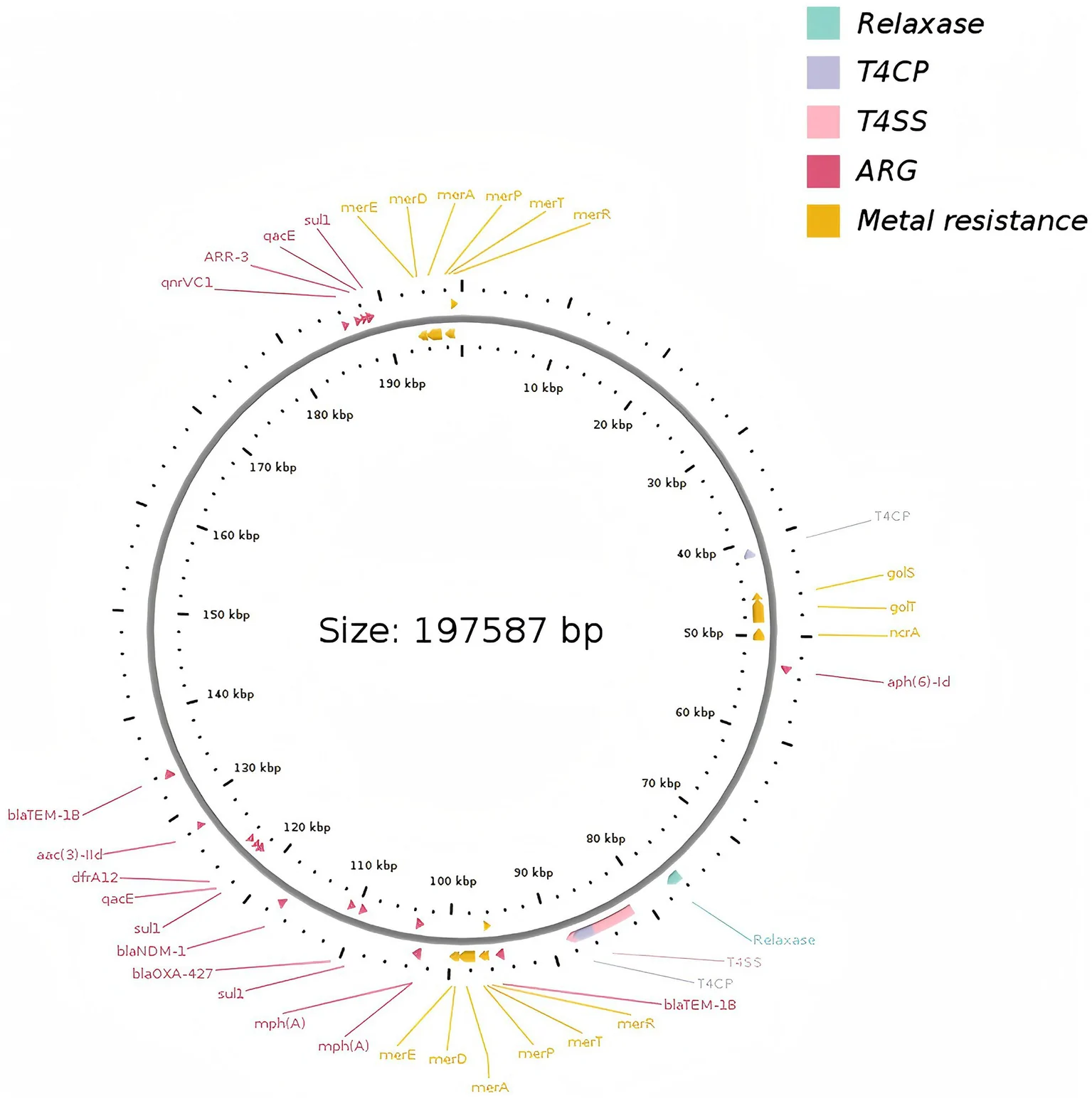
Visualization of annotation results of oriTfinder2 for *bla*_NDM-1_ resistant plasmid.

For comparative genomic analysis, two NDM-1-carrying *A. caviae* plasmids were downloaded from the NCBI database: pK433-NDM (accession No. OK287926) from strain K433 isolated from sputum in Taizhou, Zhejiang, China, and pFAHZZU2447_NDM (accession No. CP100393) from strain FAHZZU2447 isolated from bile in Zhengzhou, China. Synteny comparison of the genetic environments of the three *bla*_NDM-1_-carrying *A. caviae* plasmids was performed using Easyfig. The core module *bla*_NDM-1_-*ble*_MBL_-*trpF* was highly conserved across all three plasmids (99–100% identity), suggesting that this module is the core unit mediating horizontal transfer of *bla*_NDM-1_ in *A. caviae*. However, the flanking regions differed substantially. In the plasmid of clinical isolate LHL25108073, *bla*_NDM_ was flanked upstream by IS91-IS1, lacking the ISCR1 element present in the other two plasmids, and carried an intact *bla*_OXA-like_ gene with a *creC*-*creB* arrangement further upstream. The downstream region comprised IS91-*sul1*-*intI1*-IS15-ISAeme4, in which *intI1* was linked to *sul1* but lacked *qacEΔ1* and gene cassettes, representing an incomplete class 1 integron. In pK433-NDM (OK287926), the upstream region carried ISCR1-ΔISAba125-IS1R, and the downstream region contained a complete class 1 integron with a *dfrA12* cassette, along with a truncated Δ*sul1*. In CP100393, the upstream region comprised ISCR1-ΔIS30-Δ*sul1*-IS1B, and the downstream region contained a complete class 1 integron with a *dfrA5* cassette, together with ΔblrA and ΔTat. The integrons of the two reference plasmids carried different cassettes (*dfrA12* and *dfrA5*), reflecting dynamic cassette capture and replacement. These findings indicate that the *bla*_NDM-1_ core module can be mobilized by distinct MGE frameworks, including ISCR1, IS91, and IS family elements, and recruit accessory resistance elements, contributing to the ongoing evolution of multidrug-resistant plasmids in *A. caviae* (Figure 3).

**Figure 3:**
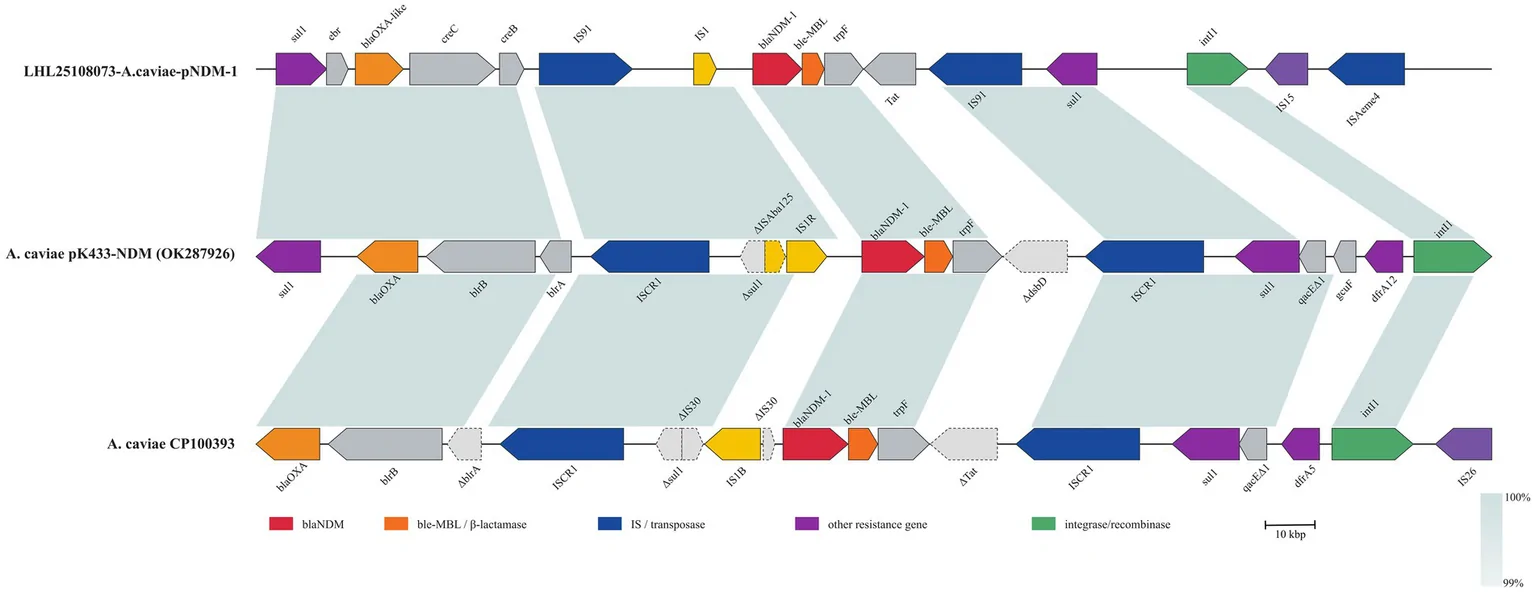
Synteny comparison of the *bla*_NDM-1_ genetic regions in three NDM-1-carrying *Aeromonas caviae pla*smids: pLHL25108073 (this study), pK433-NDM (OK287926), and pFAHZZU2447_NDM (CP100393). Arrows indicate predicted ORFs and their transcriptional orientations. Genes are color-coded by function: Red, *bla*_NDM_; orange, *ble*_MBL_/_β-lactamase_; dark blue, IS elements/transposases; purple, other resistance genes; green, integrases/recombinases; gray, other genes. Truncated genes are indicated by Δ. Gray shading between plasmids represents BLASTn identity, ranging from 99 to 100%. Scale bar represents 10 kbp.

### 3.5 Genetic background analysis

We downloaded the publicly available genome sequences of 157 *A. caviae* isolates from China and neighboring regions from the NCBI Assembly database for a genetic background analysis. These isolates were sourced from Bangladesh (50 isolates), Japan (45 isolates), China (35 isolates), Pakistan (23 isolates), and India (4 isolates), with a time span from 2015 to 2026.

Among these *A. caviae* isolates, 75 STs were identified, dominated by ST313 (29/157, 18.47%), followed by ST1093 (19/157, 12.10%). The other STs were relatively scattered, including ST829 (6 isolates), ST1252 (4 isolates), ST2281 (3 isolates), ST1250 (3 isolates), and ST1454 (3 isolates), ST1534 (3 isolates), ST819 (3 isolates), ST1581 (3 isolates), ST1491 (3 isolates), ST1932 (3 isolates), ST1263 (3 isolates), ST1321 (3 isolates), ST1095 (2 isolates), ST1251 (2 isolates), ST202 (2 isolates), ST1259 (2 isolates), ST1328 (2 isolates), ST1449 (2 isolates), ST1442 (2 isolates), ST1587 (2 isolates), and a single isolate each for ST1351, ST1436, ST1437, ST1441, ST1443, ST1445, ST1446, ST1447, ST1448, ST1450, ST1453, ST1458, ST1469, ST1476, ST1479, ST1484, ST1486, ST1489, ST1493, ST1494, ST1495, ST1496, ST1497, ST1498, ST1504, ST1505, ST1506, ST1509, ST1523, ST1524, ST1525, ST1526, ST1546, ST1547, ST1578, ST1584, ST1586, ST1898, ST2037, ST2062, ST2063, ST2085, ST2127, ST2360, ST2491, ST2682, ST592, ST645, ST823, ST831, ST1065, ST1253, and ST1268.

GrapeTree-based MLST typing revealed that 16 out of 35 Chinese *A. caviae* isolates belonged to ST313, comprising 45.71% (16/35). Meanwhile, all three identified ST2281 isolates were all of Chinese origin (8.57%, 3/35). ST2281 and ST313 isolates were connected at the allelic profile level, indicating partial similarity across their MLST housekeeping gene loci (Figure 4). Nevertheless, in the SNP-based phylogenetic tree (Figure 5) constructed where recombinant regions were removed using Gubbins, the isolate in this research was clustered together with two other publicly available ST2281 *A. caviae* reference genomes to one monophyletic clade, confirming its assignment to ST2281. This ST2281 clade did not form a closely related cluster with ST313 isolates, and the two types exhibited substantial genome-wide genetic divergence. Collectively, these findings demonstrate that the isolate characterized herein belongs to a relatively distinct ST2281 genetic lineage in the SNP-based phylogenetic tree that has rarely been documented in public databases. However, these observations are based on the limited number of genomes currently available in NCBI, which are subject to sampling bias in terms of source and geographic distribution, and the associations between STs and geography or source require further validation with larger sample sizes.

**Figure 4:**
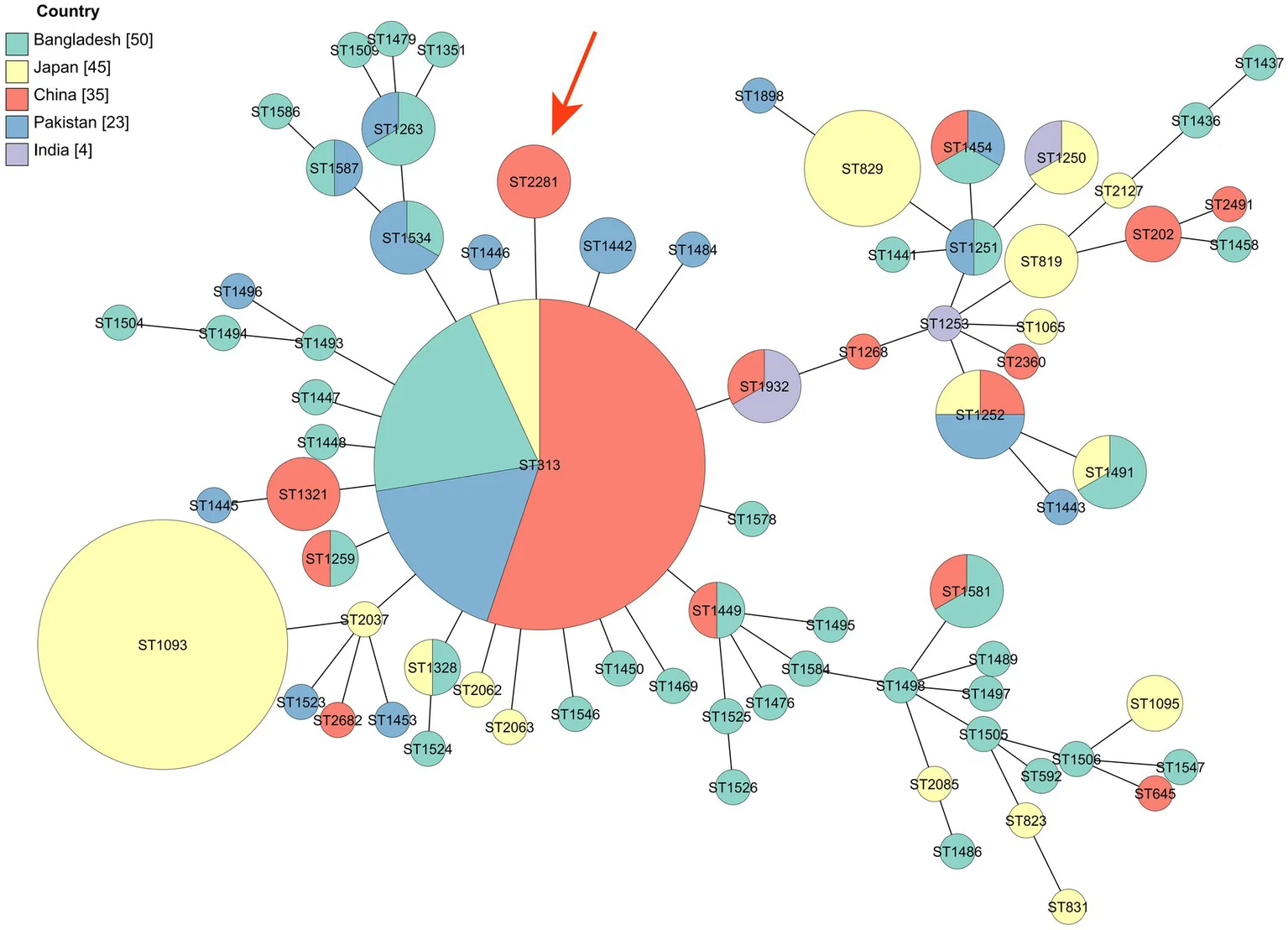
Minimum spanning tree of *A. caviae* constructed based on MLST allele profiles illustrating the geographic distribution and phylogenetic relationships of sequence types (STs) among the strains isolated in this study and those retrieved from public databases in China and neighboring regions.

**Figure 5:**
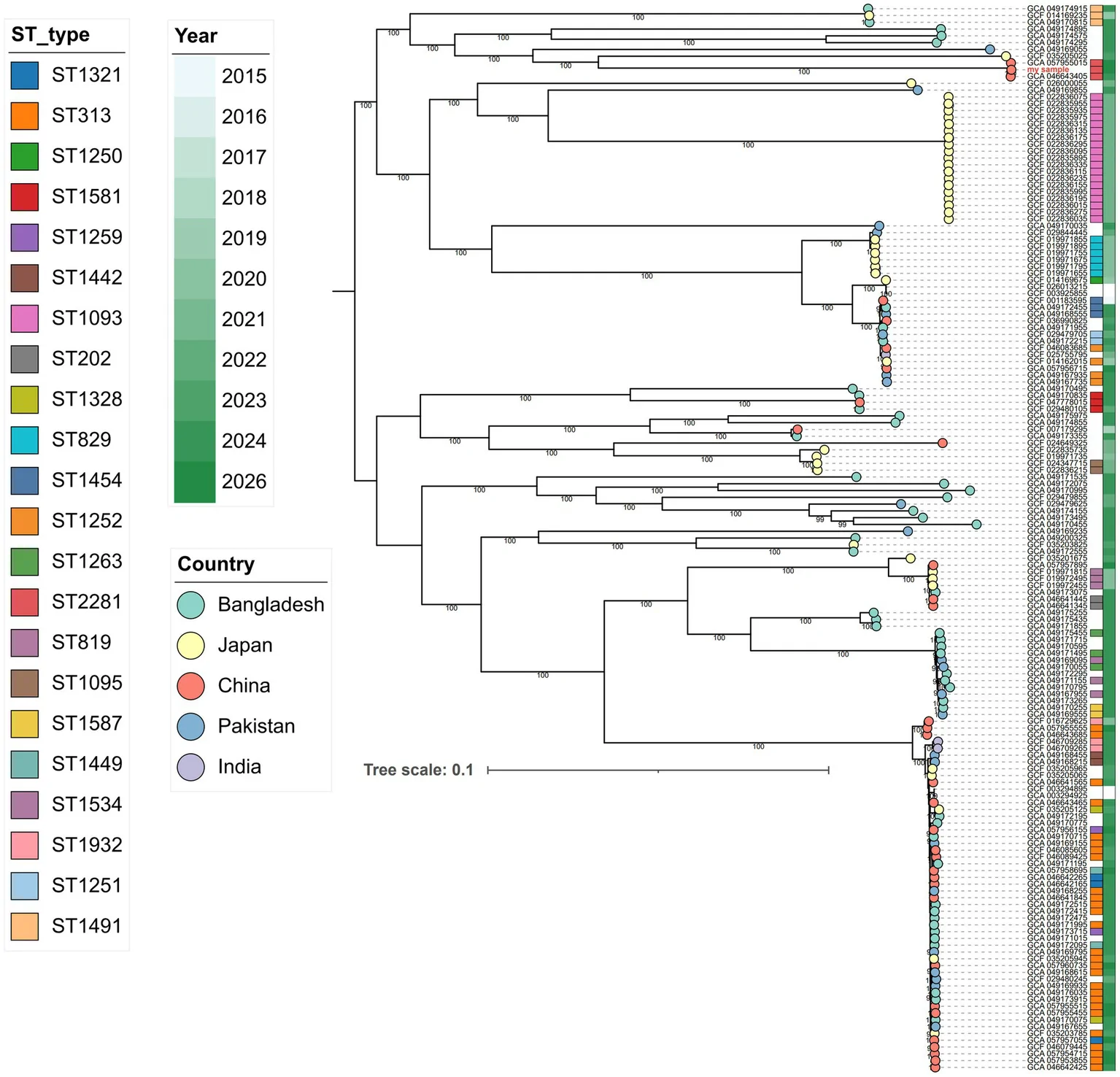
Core-genome single-nucleotide polymorphism (SNP) phylogenetic tree constructed after removal of recombinant regions using Gubbins. The isolates obtained in this study are shown in red and form a distinct subclade together with other reference genomes belonging to sequence type 2,281 (ST2281).

## 4 Discussion

This study systematically analyzed the biological characteristics of a bile-derived isolate of NDM-1-producing *Aeromonas caviae* (LHL25108073-*A. caviae*) through multidimensional analysis such as MLST typing, virulence and resistance gene annotation, whole-genome alignment, and conjugation experiments. The MLST typing results showed that the isolate was classified as the ST2281 type, and its allele combinations (*gltA*_89, *groL*_751, *gyrB*_768, *metG*_595, *ppsA*_914, *recA*_675) were relatively rare in the reported *A. caviae* typing.

The genome sequencing results revealed that the isolate contained a reservoir of virulence genes, and the detection of 58 high-confidence virulence genes was consistent with its pathogenic potential. Among them, the synergistic expression of genes related to common flagella (*fli*, *flg*, *flh* families), lateral flagella (*lfi*, *lfg*, *lfh*, *laf* families), and polar flagella (*motY*, *pomA2*) can facilitate bacterial colonization and dissemination within the biliary system by enhancing their motility (Sharma et al., 2022). This isolate also harbors genes encoding type IV pili (*tapB/C/T*) and *Msh* pili (*mshE*) (Floyd et al., 2020; Rijal and Curtis, 2026). Both types of pili can mediate stable bacterial adhesion by recognizing glycoprotein receptors on the host cell surface (Hughes et al., 2022), which significantly strengthens resistance to host clearance and represents a key molecular basis for sustained survival in the flowing biliary tract. The presence of a heme-uptake gene (*amoA*) (Balhesteros et al., 2017) indicates an iron acquisition mechanism in this isolate. As an essential trace element for bacterial growth and reproduction, iron availability determines the rate of bacterial proliferation in the infected area (Khan et al., 2024). Notably, 6 T6SS genes (*hcp*, *clpB*, *vasH*, *vasK*, *vgrG*, *vgrG1*) and 4 T2SS genes (*exeE*, *exeF*, *exeG*, *exeI*) were identified, constituting a multilayered secretion system for the isolate (Moriel et al., 2021; Tan et al., 2025). T6SS is a contractile nanomachine that delivers effector proteins into adjacent bacterial or eukaryotic cells, and in *Aeromonas* it has been implicated in interbacterial competition, host colonization, and virulence (Moriel et al., 2021). In addition, the hemolysin gene *hlyA* can directly destroy the host cell membrane (Cai et al., 2024). Furthermore, the chromosome harbored the conserved lipopolysaccharide (LPS) inner-core biosynthesis gene *waaF*, which encodes ADP-L-glycero-D-manno-heptose: LPS heptosyltransferase II and attaches the second heptose residue to the LPS inner core. An intact LPS inner core is required for outer-membrane integrity and shields Gram-negative bacteria against bile salts, serum complement, and hydrophobic antimicrobials (Maher and Hassan, 2023; Sidor and Skirecki, 2023), which may facilitate persistence of this isolate in the bile-salt-rich biliary tract. Full-length *waaF* homologs were conserved in all nine comparison genomes with retained upstream synteny, indicating that *waaF* is a stably inherited core fitness and virulence determinant of *A. caviae* rather than a horizontally acquired accessory element. The synergistic effect of these virulence genes explains the molecular mechanism of severe infection caused by this isolate and also underscores the importance of integrating both anti-infection and anti-inflammatory interventions into clinical treatment.

The analysis of drug resistance genes showed that the isolate in this study carried 14 drug resistance genes and exhibited a multidrug resistance phenotype, and the universality and genetic diversity of its drug resistance spectrum are worrying. The carbapenem resistance gene NDM-1, the core resistance determinant of this isolate, conferred resistance by hydrolyzing the β-lactam ring structure of carbapenems. The antimicrobial susceptibility results showed that this isolate was resistant to most β-lactam agents but only sensitive to amikacin, aztreonam, ciprofloxacin, and levofloxacin, consistent with the antimicrobial susceptibility phenotype profiles of clinical carbapenem-resistant bacteria. The plasmid also carried multiple acquired resistance genes, including β-lactamases (TEM-1, TEM-171), *sul1* conferring sulfonamide resistance, *mphA* and *mrx* conferring macrolide resistance, *dfrA12* conferring trimethoprim resistance, *arr-2* conferring rifampicin resistance, APH(6)-Id conferring streptomycin resistance, QnrVC6 conferring quinolone resistance (Tang and Zhao, 2023), and *qacEΔ1* conferring disinfectant resistance. The co-carriage of these resistance genes on a single plasmid highlights the potential for co-selection and dissemination of multidrug resistance (Baker-Austin et al., 2006). Of note, OXA-504 is a species-specific chromosomal class D β-lactamase intrinsic to *A. caviae*, analogous to OXA-12 in *A. hydrophila*, and is not associated with horizontally acquired resistance (Calvo Sánchez et al., 2025). The plasmid-borne *arr-2* and *dfrA12* genes, together with *qacEΔ1* and *sul1*, represent characteristic components of class 1 integron cassette arrays, which can capture and express diverse resistance cassettes, reflecting ongoing integron-mediated gene recruitment (Piotrowska and Popowska, 2015). The BRP (MBL) gene, located immediately downstream of *bla*_NDM-1_ within the conserved core module, encodes a bleomycin-binding protein and is consistently co-transferred with *bla*_NDM-1_ (Dortet et al., 2017). The *mphA*-*mrx* macrolide inactivation cluster, previously reported in *Aeromonas hydrophila* adjacent to a class 1 integron, and APH(6)-Id conferring streptomycin resistance, further broaden the resistance spectrum of this isolate (Poole et al., 2006; Ramirez and Tolmasky, 2010). On the chromosome, MOX-3 and OXA-504 were identified as chromosomal β-lactamases, with MOX-3 representing a species-specific class C β-lactamase intrinsic to *Aeromonas* (Philippon et al., 2022). Notably, no efflux pump genes met the stringent identity threshold (≥90%), suggesting that the multidrug resistance phenotype of this isolate is primarily driven by acquired resistance genes rather than overexpression of efflux pumps.

The whole-genome alignment results revealed genetic differences between the isolate identified in this study and *A. caviae* from different regions and sources in China. In addition, the co-localization of both resistance genes and virulence factors on the chromosome implies that this isolate may adapt to clinical environments through virulence-resistance co-evolution. This co-evolution pattern is relatively rare in multidrug-resistant bacteria and merits attention in epidemiological surveillance.

The characteristic analysis of drug-resistant plasmids in this study also provides key clues for understanding the transfer mechanism of the NDM-1 gene. This drug-resistant plasmid has a full length of 197,587 bp and carries transfer-related genes such as *relaxase*, *T4CP*, and *T4SS* (Zechner et al., 2017; Kishida et al., 2025), but it cannot autonomously transfer due to the absence of the transfer initiation site *oriT* (Libante et al., 2020), which is consistent with our results of the conjugation experiment. The identification of non-conjugative plasmids suggests that the transfer of the NDM-1 gene within this isolate may rely on alternative horizontal gene transfer pathways such as transformation and transduction. Comparative genomic analysis with two other NDM-1-carrying *A. caviae* plasmids, pK433-NDM (OK287926) from sputum in Taizhou, Zhejiang, and pFAHZZU2447_NDM (CP100393) from bile in Zhengzhou, revealed that the resistance core regions were highly conserved (99–100% identity), with identical *bla*_NDM-1_ sequences, whereas the plasmid backbone regions differed substantially. The flanking MGE structures also varied: pLHL25108073 carried IS91-IS1 flanking *bla*_NDM_ and an incomplete class 1 integron downstream, while the two reference plasmids carried ISCR1 and complete class 1 integrons with *dfrA12* or *dfrA5* cassettes. These findings suggest that these plasmids may have originated from a common ancestral plasmid and subsequently undergone backbone insertions, deletions, and recombination events following geographic dispersal.

The chromosome of LHL25108073 harbored multiple mobile genetic elements, including at least three prophage regions, two of which were P2-like (approximately 91–123 kb, 846–863 kb, and 4,672–4,687 kb), numerous insertion sequences belonging to the IS3, IS4, IS630, IS1595, IS481, Tn3, and IS2 families, and several site-specific integrases and tyrosine recombinases. Notably, the chromosomally located β-lactamase gene *bla*_CMY-1/MOX-3_ was not directly flanked by recognizable MGEs within 10 kb, suggesting that it is unlikely to have been recently acquired via transposition and may represent an intrinsic or anciently integrated determinant.

Further, based on an integrative analysis of publicly available genome data from 157 *A. caviae* isolates in China and neighboring regions (mainly in the Asia-Pacific region) from 2015 to 2026, these *A. caviae* isolates were assigned to 75 distinct STs, showing a distribution pattern of dominant prevalent STs and highly scattered rare STs. Among them, ST313 is the global dominant lineage, accounting for 18.47%, ST1093 is a secondary prevalent type, and most of the remaining STs only exist in sporadic strains. Based on the genetic background analysis of domestic isolates, it was found that the ST313 type was also the most prevalent clonal lineage of *A. caviae* in China, accounting for 45.71% of all domestic isolates. All of three ST2281 *A. caviae* isolates deposited in public databases were sourced from China, making up 8.57% of the total collection. MLST allele profiling analysis showed partial housekeeping gene homology between ST2281 and the dominant ST313 isolate in China and a weak correlation between their evolutionary lineages. However, after removing the recombination region using Gubbins software and constructing a phylogenetic tree based on whole-genome SNPs, it was found that the isolate in this study was closely clustered with the other two ST2281 isolates into a single independent clade, with a significant whole-genome genetic distance from the dominant lineage ST313, without a close evolutionary relationship. This confirms that ST2281 is an independent and rare genetic lineage distinct from the mainstream ST313. Public database records reveal sparse global reports of ST2281 *A. caviae* (Song et al., 2023). Critical traits of this uncommon clonal lineage, including host adaptability, pathogenicity, and risk of resistance transmission, have not been fully elucidated (Meng et al., 2021). Therefore, this bile-derived NDM-1-producing ST2281 *A. caviae* isolate not only enriches the genome data of this rare lineage but also suggests the emergence of carbapenem-resistant variants within a previously underexplored clone in China. This study supplements key data for elucidating the evolutionary and epidemiological characteristics of *A. caviae* drug resistance. At the same time, the hidden existence of this rare lineage also implies that a large number of undiscovered drug-resistant clones persist in clinical and environmental settings, and their evolutionary variations and cross-host transmission risks cannot be ignored. Continuous surveillance is urgently required to guard against clinical infections and local epidemics caused by rare clones acquiring multiple drug-resistant genes.

This study is based on a single clinical isolate; the findings regarding the genomic features, resistance gene contexts, and virulence repertoire of ST2281 *A. caviae* should be interpreted accordingly, and larger-scale surveillance and functional studies are needed to validate these observations.

In summary, this study systematically reports the genome-wide characteristics of the NDM-1-producing ST2281 *A. caviae* isolate from extraintestinal bile. It is confirmed that this isolate carries 58 high-confidence virulence genes and 14 drug resistance genes, and the carried NDM-1 resistance plasmid is non-conjugative. These findings imply that dissemination of the resistance gene relies on non-conjugative gene transfer pathways such as transformation and transduction, posing a potential risk of drug resistance transmission. Our results underscore the urgent need for strengthened collaboration with clinical microbiology laboratories to enable prompt resistance gene genotyping. Clinicians ought to strictly select sensitive antibiotics for precise treatment and standardize the clinical application of antibiotics to delay the progressive dissemination of bacterial resistance. Moreover, close attention must be paid to the hidden epidemic of rare resistant clones in clinical and public health settings to prevent nosocomial infections and regional outbreaks stemming from the evolution and transmission of these rare resistant strains.

## 5 Conclusion

This study systematically analyzed the molecular genetics, pathogenicity, and drug resistance characteristics of clinical bile-derived LHL25108073-*A. caviae* isolate (NDM-1-producing *A. caviae*) through whole-genome sequencing, bioinformatics analysis, and *in vitro* conjugation experiments. This isolate is a rare ST2281 type in China belonging to a rare and niche lineage of independent evolution, distinct from the dominant prevalent ST313 *A. caviae* in China and neighboring regions. Regarding drug resistance, this isolate carries 14 resistance genes, with *bla*_NDM-1_ as the core resistance determinant, combined with plasmid-mediated resistance genes (*sul1*, TEM-1, TEM-171, *mphA*, *mrx*, *dfrA12*, *arr-2*, APH(6)-Id, QnrVC6, BRP(MBL), *qacEΔ1*) and chromosomal β-lactamases (MOX-3, OXA-504) to form a multi-drug resistance phenotype. The isolate is only sensitive to amikacin, aztreonam, ciprofloxacin, and levofloxacin, with a virulence-resistance co-evolution feature on its chromosome. The *bla*_NDM-1_ isolate is located on a 197,587 bp non-conjugative plasmid and cannot autonomously conjugate and transfer due to the loss of the oriT site. The horizontal transfer of resistance genes may rely on transformation and transduction. Comparative genomic analysis with two NDM-1-carrying *A. caviae* plasmids from Taizhou (pK433-NDM, OK287926) and Zhengzhou (pFAHZZU2447_NDM, CP100393) revealed highly conserved resistance core regions with identical *bla*_NDM-1_ sequences, suggesting a common ancestral origin. The study provides a theoretical basis for preventing nosocomial infections and regional outbreaks of such rare resistant strains.

## Data Availability

The datasets presented in this study can be found in online repositories. The names of the repository/repositories and accession number(s) can be found in the article/Supplementary material. The whole-genome sequence of isolate LHL25108073-A. caviae was deposited in NCBI with accession number JBSUSV000000000.

## Footnotes

1
https://pubmlst.org/species-id

2
http://www.mgc.ac.cn/VFs/

3
https://card.mcmaster.ca/

4
https://cge.food.dtu.dk/services/PlasmidFinder/
